# The Current Discussion Regarding End-of-Life Care for Patients with Out-of-Hospital Cardiac Arrest with Initial Non-Shockable Rhythm: A Narrative Review

**DOI:** 10.3390/medicina60040533

**Published:** 2024-03-25

**Authors:** Junki Ishii, Mitsuaki Nishikimi, Shinichiro Ohshimo, Nobuaki Shime

**Affiliations:** Department of Emergency and Critical Care Medicine, Graduate School of Biomedical and Health Sciences, Hiroshima University, Hiroshima 734-8551, Japan; ishii824@hiroshima-u.ac.jp (J.I.); ohshimos@hiroshima-u.ac.jp (S.O.); shime@koto.kpu-m.ac.jp (N.S.)

**Keywords:** out-of-hospital cardiac arrest, non-shockable rhythm, end-of-life care, advance directive, termination of resuscitation

## Abstract

Despite recent advances in resuscitation science, outcomes in patients with out-of-hospital cardiac arrest (OHCA) with initial non-shockable rhythm remains poor. Those with initial non-shockable rhythm have some epidemiological features, including the proportion of patients with a witnessed arrest, bystander cardiopulmonary resuscitation (CPR), age, and presumed etiology of cardiac arrest have been reported, which differ from those with initial shockable rhythm. The discussion regarding better end-of-life care for patients with OHCA is a major concern among citizens. As one of the efforts to avoid unwanted resuscitation, advance directive is recognized as a key intervention, safeguarding patient autonomy. However, several difficulties remain in enhancing the effective use of advance directives for patients with OHCA, including local regulation of their use, insufficient utilization of advance directives by emergency medical services at the scene, and a lack of established tools for discussing futility of resuscitation in advance care planning. In addition, prehospital termination of resuscitation is a common practice in many emergency medical service systems to assist clinicians in deciding whether to discontinue resuscitation. However, there are also several unresolved problems, including the feasibility of implementing the rules for several regions and potential missed survivors among candidates for prehospital termination of resuscitation. Further investigation to address these difficulties is warranted for better end-of-life care of patients with OHCA.

## 1. Introduction

Out-of-hospital sudden cardiac arrest (OHCA) remains a major concern worldwide, with 350,000 patients annually in the United States (US) [1]. Despite recent advances in resuscitation science, including high-quality chest compression, immediate defibrillation and targeted temperature management, patient outcomes remain poor; 9% of patients with OHCA achieved survival to hospital discharge, and only 7% had favorable neurological function at hospital discharge [2].

Previous studies have shown that patient outcomes differ according to the initial cardiac rhythm, and patients with OHCA with initial non-shockable rhythm are especially likely to exhibit poor survival and neurological outcomes. In addition to seeking better patient outcomes, a discussion of better end-of-life care is a major concern for citizens.

In this review, we discuss the following issues among patients with OHCA with initial non-shockable rhythm: 1. epidemiology and clinical outcomes; 2. end-of-life care issues; and 3. future studies aiming to provide better end-of-life care.

## 2. Methodology

This narrative review extracted and collected data regarding the characteristics and outcomes of patients with OHCA according to initial cardiac rhythm and issues of end-of-life care for these patients, especially advance directive (AD) and termination of resuscitation (TOR) rules. The primary aim of the study was to provide information regarding patients with OHCA with initial non-shockable rhythm and the current status of the utilization of AD and TOR rules. For this review, PubMed and Google Scholar were searched for eligible articles using combinations of the key terms “out-of-hospital cardiac arrest” or “OHCA” and “initial rhythm” or “initial cardiac rhythm” and “advance directive” or “termination of resuscitation.” The articles included in this review were published in English, and were available until February 2024.

## 3. Epidemiology and Clinical Outcomes among Patients with OHCA with Initial Non-Shockable Rhythm

According to a report from the American Heart Association, 74.7% of adult patients with OHCA have initial non-shockable rhythm (*n* = 143,284) [2]. Among them, 52.4% had initial asystole rhythm.

Patients with OHCA with initial non-shockable rhythm are known to have worse outcomes than those with initial shockable rhythm. A study using a large-scale database of 170,678 patients reported that 27.9% of patients with initial shockable rhythm achieved prehospital return of spontaneous circulation (ROSC), whereas in those with initial non-shockable rhythm, it was only 6.0% [3]. An American Heart Association report showed that the survival to hospital admission rate among patients with initial non-shockable rhythm was 20.7%, compared to 44.5% among those with initial shockable rhythm [2]. Furthermore, the survival to hospital discharge rate and good neurological outcome (Cerebral Performance Category scale of 1 or 2) were considerably poorer among those with initial non-shockable rhythm than those with initial shockable rhythm (5.7% vs. 25.9% and 4.0% vs. 22.5%, respectively) [2]. Another observational study revealed that patients with OHCA with initial non-shockable rhythm had statistically significantly lower resilience for neurologically intact survival after prolonged periods of resuscitation than those with initial shockable rhythm [4], and that initial non-shockable rhythm was an independent predictor of the worse outcome [5].

Some epidemiological differences that can explain worse outcomes of patients with OHCA with initial non-shockable rhythm have also been reported in previous studies. For example, a study from Canada reported that 69% of patients with initial shockable rhythm were witnessed, whereas only 31% of those with initial non-shockable rhythm were witnessed [4]. In a large-scale study that included 29,863 patients from Denmark, 76.9% of patients with initial shockable rhythm were witnessed arrest, whereas only 42.6% of those with initial non-shockable rhythm had a witnessed arrest [6]. The proportion of patients who received bystander cardiopulmonary resuscitation (CPR) also differed; 54.9% and 30.7% of patients had initial shockable and non-shockable rhythms, respectively [6]. Age may also account for a higher proportion of poorer outcomes for patients with OHCA with initial non-shockable rhythm; various studies have shown differences in the median age of patients with OHCA with initial non-shockable and shockable rhythms [4,6,7]. In a report from Canada, the median age of patients with OHCA with initial non-shockable rhythm was 71 years (interquartile range [IQR]: 55–82), whereas that of patients with OHCA with initial shockable rhythm was 64 years (IQR: 54–75) [4]. Similar results were reported in a study in Denmark (71 [IQR: 60–81] with initial non-shockable rhythm vs. 67 [IQR: 58–77] with initial shockable rhythm) [6] and data from Australia and the United Kingdom (71 [IQR: 51–97] with initial non-shockable rhythm vs. 69 [IQR: 47–91] with initial shockable rhythm) [7]. The presumed etiology of cardiac arrest differs between patients with initial non-shockable and shockable rhythms. Previous studies have shown that the proportion of patients with cardiac etiology as cardiac arrest was relatively lower among those with initial non-shockable rhythm compared to those with initial shockable rhythm: 70% vs. 93% in a study from Denmark [6] and 40% vs. 97% in a study from the US [8]. Considering the relatively poorer outcomes among patients with OHCA with a non-cardiac etiology than among those with a cardiac etiology [9], this difference may also be associated with poorer outcomes among those with initial non-shockable rhythm. The characteristics and outcomes of patients with OHCA with initial non-shockable and shockable rhythms from the literature examined in this study are summarized in Table 1.

In summary, compared with those with initial shockable rhythm, patients with OHCA with initial non-shockable rhythm tend to be older, have less witnessed arrest, and receive less bystander CPR. These factors may contribute to relatively poorer outcomes among patients with initial non-shockable rhythm than patients with initial shockable rhythm.

## 4. End-of-Life Issues among Patients with OHCA with Initial Non-Shockable Rhythm

**Figure 1:**
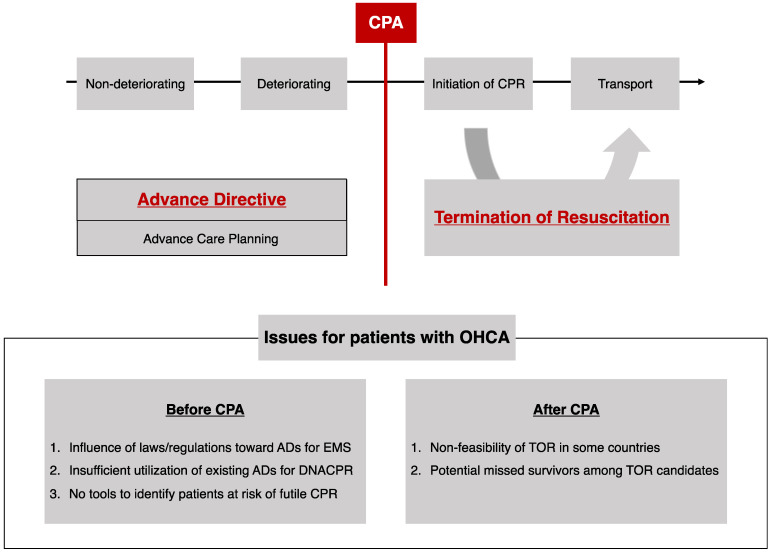
Issues in pursuing better end-of-life care for patients with OHCA. OHCA, out-of-hospital cardiac arrest; CPA, cardiopulmonary arrest; CPR, cardiopulmonary resuscitation; AD, advance directive; EMS, emergency medical services; DNACPR, do-not-attempt cardiopulmonary resuscitation; TOR, termination of resuscitation.

Considering that many patients with OHCA with initial non-shockable rhythm show poor neurological outcomes, how to prepare for cardiac arrest and to provide better end-of-life care for patients with OHCA with initial non-shockable rhythm is worth discussing. In this section, we discuss the issues that impede achieving a better end-of-life, before and after CPR (Figure 1).

### 4.1. Before CPR

As sudden cardiac arrest can occur unexpectedly, and CPR may be attempted on unwilling patients, the importance of the effort to avoid unwanted resuscitation has been gradually recognized globally. The European Resuscitation Council (ERC) Guidelines 2021 were published to provide evidence-based recommendations for the ethical, routine practice of resuscitation and end-of-life care. The guidelines introduced AD and advance care planning as key interventions that safeguard patient autonomy, even in situations where patients are unable to articulate their preferences regarding treatment decisions [10,11,12,13,14]. While an AD is a document that communicates information about an individual’s preferences and goals for medical procedures and treatments, advance care planning is a process that enables individuals to define and discuss goals and preferences for future medical treatment and care with family and healthcare professionals, including documenting their preferences as ADs. At the scene of resuscitation, a document (e.g., AD) provides essential information regarding patient preferences.

Not surprisingly, for patients with OHCA, having an AD for do-not-attempt CPR (DNACPR) is critical in avoiding needless invasive medical procedures, including chest compression and defibrillation. According to recent studies, AD for DNACPR and/or DNACPR decisions before cardiac arrest were associated with a reduced use of life-sustaining treatment [13]. However, several difficulties remain in enhancing the effective use of ADs for patients with OHCA.

#### 4.1.1. Influence of Regional Laws and/or Administrative Regulations on ADs for Emergency Medical Services

In a scenario where ADs accurately reflect the patient’s wishes and have been made in accordance with consensus definitions and statements [13], ADs should be utilized without regulation to safeguard patient autonomy, which is a primary objective of the ADs [13]. However, there is considerable regional variation in the utilization of ADs for patients with OHCA, and some countries cannot utilize ADs effectively because of local laws and/or administrative regulations. For example, while in the US and several European countries, emergency medical services are usually trained to forgo resuscitation if there is a formal AD [15,16], emergency medical services in Japan are generally restricted from deciding not to start resuscitation, by administration, even if victims have written or verbal ADs. A previous Japanese study reported that 95.6% of patients who had DNACPR orders in advance underwent resuscitation by emergency medical services, and 37.8% were transported to a tertiary emergency hospital [17], indicating the difficulty of effective use of ADs under restricted utilization.

#### 4.1.2. Insufficient Utilization of Existing ADs for DNACPR at the Scene

Even in areas where ADs are legally or administratively recognized and effectively utilized by emergency medical services, there are cases where unwanted CPR was performed. Counts et al. reported that in a US cohort of 3152 patients with OHCA who were attended to by emergency medical services personnel, 9.9% had DNACPR directives, and approximately 30% of patients with ADs received CPR despite DNACPR directives, including approximately 6% with full advanced cardiovascular life support [15]. Another report from France showed that emergency medical services personnel accessed ADs for 7.5% of patients with OHCA, and 24% of patients whose ADs were accessed by emergency medical services personnel received CPR [16]. The reasons for these results, such as no in-hand ADs at the scene of resuscitation and family refusal of ADs [15], warrant further investigation.

#### 4.1.3. No Established Tools to Identify the Patients at Risk of Potential Futile Resuscitation

One of the situations for discussing DNACPR decisions in advance care planning is when CPR is judged very unlikely to be effective to achieve the survival, neurological, or functional outcomes anticipated by the patient, due to the patient’s condition [18]. Although providing objective information for patients is essential in the process of documenting ADs, there are no established tools to predict those at risk of “futile” CPR before they suffer OHCA with high accuracy, which is also a significant issue. Few studies have investigated how to predict the futility of resuscitation before an individual experiences OHCA. Only one brief report implied that the Supportive and Palliative Care Indicators Tool (SPICT), originally derived for identifying people with deteriorating health due to advanced conditions or a serious illness [19], can be a candidate tool for identifying those with no chance of survival after OHCA before cardiac arrest. In this retrospective observational study, 283 patients with OHCA were stratified into three risk groups for lack of survival according to SPICT scores; only 2.5% of those among the intermediate-high-risk groups survived to discharge [20]. However, considering the small sample size of this study, and the lack of statistical investigation of the prediction accuracy, further studies are required to implement this into real-world practice. In relation to this topic, difficulty in defining “futility” of resuscitation is another concern. Medical futility has been discussed in various fields, including resuscitation science [21], and the balance between the benefits and harms of resuscitation should be carefully evaluated from the individual patient’s perspective, with their autonomy [22]. Ideally, various tools to predict different outcomes should be established to support individual decision-making according to diverse preferences.

### 4.2. After CPR

The decision to stop CPR is a key component of end-of-life care issues after the initiation of CPR. Considering the disadvantages of providing full resuscitation efforts to patients with no chance of achieving ROSC (including increasing risk of hazards to emergency medical services personnel, limiting the availability of emergency medical services to care for other patients, reducing emergency department and hospital resources (including beds and equipment)), derivation of termination of resuscitation (TOR) guidelines to predict the futility of a continuing resuscitation effort have been attempted as early as the 1980s [23,24,25,26,27].

A study from Canada derived the basic life support TOR (BLS-TOR) rule for emergency medical technicians, which showed a sensitivity of 100% for identifying survival at hospital discharge and a negative predictive value of 100% for identifying non-survival at hospital discharge [28]. This rule classified those who met the following three criteria in the prehospital setting as candidates for TOR: no ROSC, no automated external defibrillator used or manual shock applied in an out-of-hospital setting, and no witness of arrest by emergency medical services personnel. This rule was subsequently validated in a second cohort of 1240 patients with OHCA with a specificity of 90.2% for recommending transport of survivors to the hospital and had a positive predictive value of 99.5% for death when termination was recommended [29]. By implementing this rule, the proportion of transportation to hospitals decreased from 100% to 37.4% [29].

In 2002, a more conservative advanced life support TOR (ALS-TOR) rule was derived from over 4000 patients with two additional criteria: no bystander-witnessed arrest and no bystander CPR. This rule resulted in no survivors at hospital discharge among those who met all five criteria and transport reduction from 100% to 70% [30]. Through external validation and comparison of the two TOR rules on the same 2415 patients, a previous study concluded that implementing BLS-TOR as a universal TOR rule would result in a lower overall transport rate without missing potential survivors [31].

A systematic review in 2020 identified 15 TOR rules, where the BLS-TOR and ALS-TOR rules were reported to be the most externally validated. The use of these TOR rules is recommended to assist clinicians in deciding whether to discontinue resuscitation efforts out of hospital in the latest resuscitation guidelines [32]. Although TOR is acknowledged as a common practice in many medical services systems [32], several issues regarding prehospital TOR rules have been discussed.

#### 4.2.1. Barriers for the Use of TOR Rules Due to Legal, Administrative, and Cultural Aspects

First, implementing TOR rules may not be feasible in some countries due to legal, administrative, and cultural aspects. For example, in Japan, TOR by emergency medical services personnel in prehospital settings is administratively prohibited [33], except in cases that satisfy all of the following requirements: patients are in terminal stage, have ADs not to resuscitate, patients’ families do not wish to perform CPR, and physicians in charge instruct emergency medical services personnel to terminate CPR in direct communication. CPR is also not performed in cases where death is certain, such as decapitation, incineration, decomposition, rigor mortis, or dependent cyanosis [34]. Pronouncement of death is made by physicians who, in Japan, have the sole authority to pronounce death and issue official death certificates. Cultural aspects among citizens expect physicians to pronounce death, on site, after a certain effort to treat the victims. These factors may explain why prehospital TOR rules are not utilized in Japan; however, further studies are warranted. In addition, in Japan, in-depth end-of-life care discussions on emergency and critical care medicine only started in the mid-2010s [34], which is relatively more recent than in North America or Europe, which may also affect the difference in implementation status of prehospital TOR rules among the regions.

#### 4.2.2. Potential Missed Survivors among TOR Candidates

Second, some patients with OHCA were classified as TOR candidates, based on the rules, but survived. In a validation study of the BLS-TOR rule, four (0.5%) patients survived to hospital discharge among those who met the BLS-TOR rule, although the predictive accuracy improved when either emergency medical services personnel response time or absence of a witness was incorporated [29]. Similarly, a large-scale study of 5505 patients in the US showed that the BLS-TOR rule classified five (0.2%) surviving patients as prehospital TOR candidates [35]. The fact that current TOR rules cannot perfectly predict their prognosis is one of the major ethical issues, and discussions on the acceptable threshold of potentially missed survival rates are required [21,22].

In summary, while TOR is acknowledged as common practice in many EMS systems, issues still exist regarding the non-feasibility of implementing TOR rules due to local laws, administrations, cultural factors and potential missed survivors among TOR candidates.

## 5. Future Work to Achieve Better End-of-Life Care among Patients with OHCA with Initial Non-Shockable Rhythm

### 5.1. Before CPR

#### 5.1.1. Influence of Regional Laws and/or Administrative Regulations toward ADs for EMS Services

In regions where regulations regarding ADs for emergency medical services exist, future studies are required to explore the reasons for these regulations. Studies comparing the regulatory status in different countries would also be useful for identifying the reasons for the insufficient utilization of ADs in emergency medical services. The development of an international database, including information on the presence of ADs and the prevalence of advance care planning in different countries, may be valuable for comparative studies. Additionally, barriers and facilitators to implementing ADs from the perspective of non-medical stakeholders, such as lawyers and politicians, warrant examination. Although prudent discussion with multifaceted stakeholders is necessary for the practical utilization of ADs at the scene, a recent integrative review of 93 studies found no studies examining the views and experiences of legal professionals in developing advance care planning and ADs [36].

#### 5.1.2. Insufficient Utilization of Existing ADs for DNACPR at the Scene

Studies investigating the reasons for emergency medical service activation by bystanders, despite ADs for DNACPR, should be encouraged. Counts et al. assumed that this might be due to bystander uncertainty regarding end-of-life preferences, requests for care other than resuscitation, or the need to confirm death, even if DNACPR status is understood [15]. With these future studies, further research to evaluate and implement a novel method to enhance the effectiveness of ADs for DNACPR at the scene will become available. For example, dispatch guidance may be a potential solution to this problem. Developing a registry that provides real-time access to identify and/or confirm a person’s DNACPR status would also be helpful in facilitating the utilization of existing ADs for DNACPR. It may also be valuable to provide enlightenment to citizens—for example, via public lectures by emergency medical services personnel—to enhance utilization of ADs.

#### 5.1.3. No Established Tools to Identify the Patients at Risk of Potential Futile Resuscitation

Further studies are warranted to establish tools for identifying the patients at risk of potential futile resuscitation. As mentioned above [20], SPICT has the potential to identify patients at risk of futile resuscitation; however, to establish this, studies of statistical investigation of predictive accuracy and validation with larger sample sizes are required. As SPICT consists of qualitative components, the development of novel, more objective scoring systems may also be required. In addition, considering the variety of individuals’ perspectives regarding the balance of benefits and harms of resuscitation; these tools are warranted to identify several outcomes, such as survival, favorable neurological outcome, and favorable functional outcome.

### 5.2. After CPR

#### 5.2.1. Barriers for the Use of TOR Rules Due to Legal, Administrative, and Cultural Aspects

In addition to ADs, the implementation status of prehospital TOR rules and their barriers and facilitators should be examined in other regions. In local, legal, administrative, and cultural contexts, there may be regions where prehospital TOR rules are difficult to implement. In the new implementation of prehospital TOR rules, care should be taken for barriers, including legislation and advocacy, as shown in a previous study from the US [37]. Future qualitative documentation of barriers and discussion processes will be useful for such regions. In addition, real-world implementation trials of prehospital TOR rules may also be helpful for discussing their future implementation; the latest guidelines revealed that only one real-world implementation study [38] was detected in a systematic review [32]. In the future real-world implementation studies, outcome measurements should include the assessment of reasons among the cases in which the TOR rules are not followed, as well as compliance rate for the TOR rules, transport rate, and effects on healthcare providers involved, such as comfort level. In addition, the effects on the activities of emergency medical services personnel, such as changes in on-site activity time and the number of activations, should be evaluated. Furthermore, studies evaluating the cost-effectiveness of implementing TOR rules are worth discussing. To the best of our knowledge, only one study has evaluated the cost-effectiveness of practices with and without TOR rules for OHCA [33]. In this study, implementing the BLS-TOR rule was more cost-effective than when the ALS-TOR rule or no TOR rule was used. Although the results of the study were robust, care should be taken to implement the results in other regions or situations because costs and treatment effectiveness for patients with OHCA depends on demographics and healthcare systems. Future studies evaluating cost-effectiveness of TOR rules from various regions are warranted for a constructive discussion on the utilization of TOR rules.

#### 5.2.2. Potential Missed Survivors among TOR Candidates

Studies to improve the predictive accuracy of the current TOR rules are vital. It may also be effective to develop more specific scores, such as those focusing on patients with initial non-shockable rhythm. However, this can potentially decrease its practicability. Also, we should be aware that there are no “zero” risks. It is worthwhile for clinicians that some academic organizations illustrate thresholds, cut-offs, or frameworks to determine the “futility” of resuscitation; however, it should be modified by local, cultural, legal or administrative factors.

## 6. Limitations

Our study has several limitations. First, we did not perform a systematic review of this topic. In the future, it would be worthwhile to further investigate the current status of issues regarding end-of-life care and their potential solutions for patients with OHCA with initial non-shockable rhythm. Second, we focused only on AD and TOR regarding issues surrounding end-of-life discussions among patients with OHCA with initial non-shockable rhythm. As we recognize the general complexity surrounding end-of-life discussions among those patients, we focused on providing information about one of each issue before and after CPA (AD and TOR) to make the discussion as clear as possible. As the latest guidelines regarding the ethics of resuscitation and end-of-life care suggest, other issues warrant investigation in the future, such as advance care planning, shared decision making, communication skills, and education for patients, the public, and healthcare professionals [13]. Third, we did not fully discuss the situation when ADs should not be followed. These include situations in which there is compelling evidence that the patient may have changed their mind since completing the AD, did not understand the nature of the AD, or did not have freedom of choice at the time of drafting the AD. The situation where ADs call for an action prohibited by the regions’ laws and/or regulations is also an appropriate occasion for ADs not to be utilized [13]. Although we have discussed the current issues regarding ADs under the assumption that they are made in accordance with the consensus definition and statements to make our discussion as clear as possible, future consideration of how to manage these unfavorable situations is also worthwhile to enhance effective utilization of ADs.

## 7. Conclusions

End-of-life issues for patients with OHCA are currently spreading to resuscitation care. The utilization of ADs and TOR rules varies among regions due to legal, administrative, and cultural factors, and their effectiveness has not yet been fully established. Future studies addressing these issues should assess the detailed implementation status of these concepts across various regions, identify potential barriers for improvement, and enhance their effectiveness to provide better end-of-life care for these patients.

## Figures and Tables

**Table 1:** Characteristics and outcomes among OHCA patients with initial non-shockable and shockable rhythm.

	Reference	Non-Shockable	Shockable	Total *n*	Region
Characteristics					
Witnessed arrest, %	[4]	31.2	68.6	1617	Canada
	[6]	42.6	76.9	29,863	Denmark
Bystander CPR, %	[6]	30.7	54.9	29,863	Denmark
Age, median (IQR)	[4]	71 (55–82)	64 (54–75)	1617	Canada
	[6]	71 (60–81)	67 (58–77)	29,863	Denmark
	[7]	74 (51–97)	69 (47–91)	7848	Australia, UK
Presumed cardiac cause, %	[6]	69.2	93.4	29,863	Denmark
	[8]	40.3	96.7	5958	US
Outcomes					
ROSC, %	[3]	6.0	27.9	170,678	Asia
Survival to admission, %	[2]	20.7	44.5	142,993	US
Survival to discharge, %	[2]	5.7	25.9	142,993	US
	[5]	0.4–7.4	14.8–23.0	142,740	*
Favorable neurological outcome, %	[2]	4.0	22.5	142,993	US

* Meta-analysis. OHCA, out-of-hospital cardiac arrest; CPR, cardiopulmonary resuscitation; IQR, interquartile range; ROSC, return of spontaneous circulation; UK, the United Kingdom; US, the United States.

## Data Availability

All data derives from other studies. No original data are available from the corresponding author.
